# Effect of GABA_B_ Receptor Antagonist (CGP35348) on Learning and Memory in Albino Mice

**DOI:** 10.1155/2014/983651

**Published:** 2014-01-19

**Authors:** Quratulane Gillani, Shahid Iqbal, Fatima Arfa, Saba Khakwani, Atif Akbar, Asmat Ullah, Muhammad Ali, Furhan Iqbal

**Affiliations:** ^1^Zoology Division, Institute of Pure and Applied Biology, Bahauddin Zakariya University, Multan 60800, Pakistan; ^2^Department of Statistics, Bahauddin Zakariya University, Multan 60800, Pakistan; ^3^Department of Zoology, Quaid-I-Azam Campus, Punjab University, Lahore 54000, Pakistan; ^4^Institute of Molecular Biology and Biotechnology, Bahauddin Zakariya University, Multan 60800, Pakistan

## Abstract

The present study was designed to demonstrate the potential effect of CGP 35348 (GABA_B_ receptor antagonist) on the learning, memory formation, and neuromuscular coordination in albino mouse. Mice were intrapertoneally injected with 1 mg CGP 35348/mL of distilled water/Kg body weight, while the control animals were injected with equal volume of saline solution. A battery of neurological tests was applied following the intrapertoneal injections. Results of rota rod indicated that CGP 35348 had no effect on neuromuscular coordination in both male (*P* = 0.528) and female (*P* = 0.125) albino mice. CGP 35348 treated females demonstrated poor exploratory behavior during open filed for several parameters (time mobile (*P* = 0.04), time immobile (*P* = 0.04), rotations (*P* = 0.04), and anticlockwise rotations (*P* = 0.038)). The results for Morris water maze (MWM) retention phase indicated that CGP 35348 treated male mice took shorter latency to reach the hidden platform (*P* = 0.04) than control indicating improved memory. This observation was complemented by the swim strategies used by mice during training days in MWM as CGP 35348 treated males used more direct and focal approach to reach the platform as the training proceeded.

## 1. Introduction

Glutamate and gamma-aminobutyric acid (GABA) are among the most abundant neurotransmitters in our central nervous system. Glutamate play role in excitatory responses while the GABA act as inhibitory stimuli [1]. Approximately 30% of neurons in the brain produce GABA and almost every neuron can respond to GABA [2].

Two pharmacologically and molecularly distinct GABA receptors have been identified, GABA_A_ and GABA_B_. GABA_B_ receptors are heterodimeric G protein-coupled sites, located both pre- and postsynaptically [3, 4]. GABA_B_ receptors are widely used in the treatment of neurologic and psychiatric disorders including absence seizures and gamma-hydroxybutyrate toxicity and more recently used for the treatment of autoimmune limbic encephalitis [5]. Activation of GABA_B_ receptors produces anesthetic effects in animals with neuropathy and chronic inflammation [6]. GABA_B_ antagonists have antidepressant activity [1], cognition improvement [7–9], through inhibition of memory suppressor mechanisms [10, 11], and beneficial effects in rat models of absence epilepsy [12]. 3-Aminopropyl diethoxymethylphosphinic acid (CGP35348) is GABA mimetic containing a phosphinic acid moiety which is a centrally active blocker of GABA_B_ receptors. CGP35348 was the first GABA_B_ antagonist discovered which is able to cross blood brain barrier and is the most active GABA_B_ receptor antagonist *in vivo* [13]. The interaction of CGP35348 with other receptors appears to be negligible making it a selective GABA_B_ receptor antagonist [14]. Present study was designed to suppress the GABA_B_ receptor mediated activity by using CGP 35348 (GABA_B_ receptor antagonist) and to observe its potential effect on the learning, memory formation, and neuromuscular coordination in albino mouse.

## 2. Material and Method

### 2.1. Subjects

Eight-week old albino mice (*N* = 40, male and female 20 each) were used during these experiments. Mice were maintained in cages filled with wood chips at the core animal facility of Bahauddin Zakariya University, Multan. Mice were fed on standard rodent diet and water ad libitum and housed in individual cages. Room temperature was maintained at 22 ± 1°C.

### 2.2. Chemical

On the 20th day of life, mice were separated from their parents and fed on normal mouse diet until 9th week of life when they received intraperitoneal injections of GABA_B_ receptor antagonist (CGP35348, 3-Aminopropyl diethoxymethyl phosphinic acid) or saline solution for 5 days at the rate of 1 mg/1 mL solvent/Kg body weight/day 30 minutes prior to behavioural testing.

### 2.3. Rota Rod

The rota rod appartaus test balance and coordination and comprised of a rotating drum which rotated at the speed of 40 rpm. The time at which each animal fell from the drum was recorded. Each animal received three pretraining trials. Subsequently, each mouse completed three more consecutive trials and the longest time on the drum was used for analysis [15].

### 2.4. Open Field (OF)

Mice were observed using a video monitoring system consisting of a video camcorder coupled to computational tracking system (Any-Maze, USA). Standard parameters for locomotors activity (i.e., total distance covered, average speed, amount of large movement, amount of local movement, resting time, and frequency of spontaneous change in direction) and exploratory behaviour (i.e., rearing, crossing the center, and time spent in the margin) recorded [16].

### 2.5. Morris Water Maze (MWM)

MWM consists of a circular pool (122 cm diameter, 76 cm deep) in which mice were trained to escape from water by swimming to a hidden platform (1.5 cm beneath water surface) whose location could be identified using distal extra-maze cues attached to the room walls. Visual cues had different colors and dimensions and kept constant during the whole experiment.

The pool was divided into four quadrants (compass locations, NE, NW, SW, and SE) by a computerised tracking/image analyser system coupled to computational tracking system. The platform was placed in the middle of the NE quadrant and remained at the same position during the whole experiment.

The spatial acquisition phase consisted of 16 training trials and 4 training trials per day for 4 days with an intertrial interval of 15 min. Mice were released randomly with their heads facing the pool wall from the four compass locations, and allowed to swim and search for the platform for 120 s. When mice did not locate the platform within 120 s, animals were manually placed on the platform and allowed to remain on it for another 30 s.

On day 5, after the acquisition phase, subject received a probe trial, in which the platform was removed. Mice were released from the south start point and were allowed to swim freely for 60 s. Parameters like time and path length to reach the platform area, number of times crossing the platform area, swimming strategies to reach platform area and swimming speed were recorded [17].

### 2.6. Statistical Analysis

All data are expressed as mean ± standard deviation. For all the studied parameters of open field, rota rod MWM training, and probe trial parameters, two-sample *t*-test was applied to compare the results between treated male and females and their respected controls.

## 3. Results

### 3.1. Rota Rod

**Figure 1:**
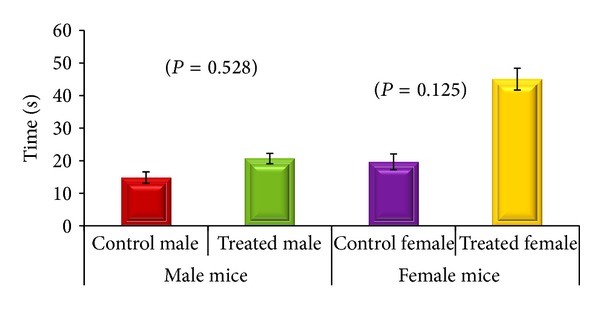
Comparison of rota rod test results control male/female with their respective GABA_B_ receptor antagonist (CGP 35348) treated male and female albino mice. Data is expressed as mean ± standard deviation. *P* values indicate the results of two-sample *t*-test.

Results indicated that the CGP 35348 injection did not affect the neuromuscular coordination in male albino mice (*P* = 0.528), but female mice treated with GABA_B_ receptor antagonist performed better on rotating rod than saline treated female albino mice, and the difference in performance did not reach the statistical significance (*P* = 0.125) (Figure 1).

### 3.2. Open Field

There were interesting gender specific results regarding the open field test as all the studied parameters remained insignificantly different when compared between CGP 35348 and saline treated male mice (data not shown here). On the other hand, CGP 35348 treated females had demonstrated poor exploratory behavior as compared to saline treated females for several parameters (time mobile (*P* = 0.04), time immobile (*P* = 0.04), rotations (*P* = 0.04), anticlockwise rotations (*P* = 0.038)) (Table 1).

### 3.3. Morris Water Maze (MWM)

**Figure 2:**
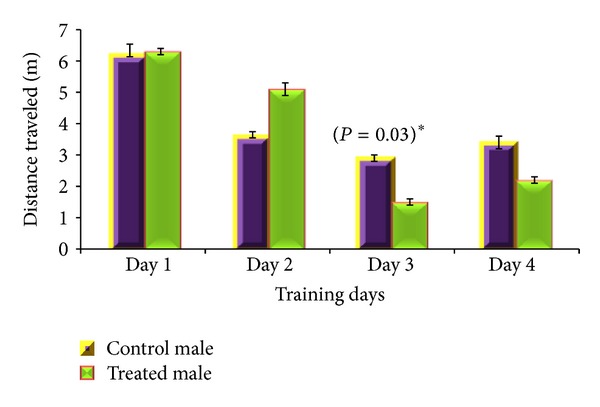
Comparison of total distance (m) travelled by GABA_B_ antagonist and saline treated male albino mouse during acquisition phase of Morris water maze test. Data is expressed as mean ± standard deviation. *P* value indicates the result of 2-sample *t*-test for the specific training day.

**Figure 3:**
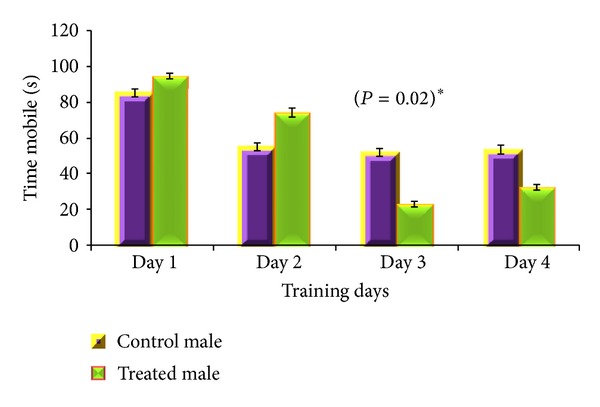
Comparison of time mobile (sec) by GABA_B_ antagonist and saline treated male albino mouse during acquisition phase of Morris water maze test. Data is expressed as mean ± standard deviation. *P* value indicates the result of 2-sample *t*-test for the specific training day.

**Figure 4:**
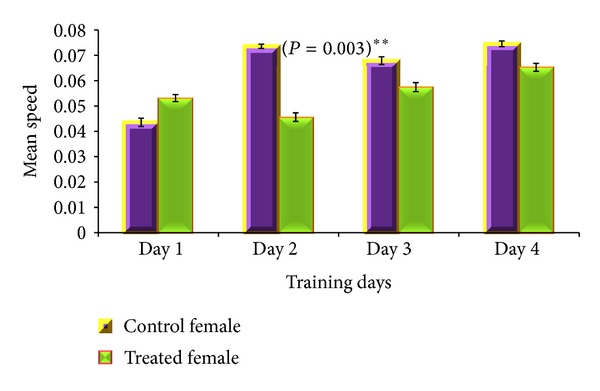
Comparison of mean speed by GABA_B_ antagonist and saline treated female albino mouse during acquisition phase of Morris Water Maze test. Data is expressed as mean ± standard deviation. *P* value indicates the result of 2-sample *t*-test for the specific training day.

Acquisition phase of MWM revealed gender specific results. For male albino mouse, total distance travelled (*P* = 0.03) and total time mobile (*P* = 0.02) were significantly different between CGP 35348 and saline treated albino mice (Figures 2 and 3) with saline treated male mice remained active for longer time and covered more distance in MWM than the GABA_B_ receptor antagonist treated male albino mice. For female mice, mean speed was the only parameter which significantly varied (*P* = 0.003) between saline and CGP 35348 treated females during training day 2 with control females swimming with more speed than treated ones (Figure 4), while all other studied parameters, for both male and female, remained insignificantly different between control and treated animals.

**Figure 5:**
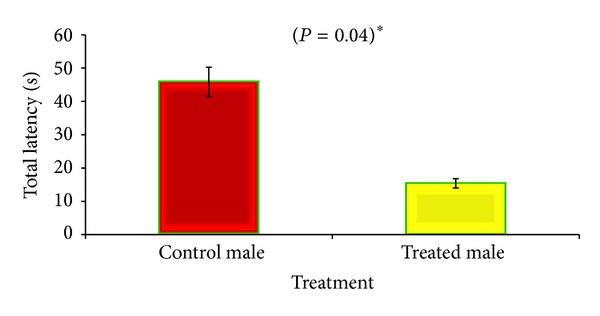
Comparison of total latency between GABA_B_ antagonist and saline treated male albino mouse during probe trial of Morris Water Maze test. Data is expressed as mean ± standard deviation. *P* value indicates the result of 2-sample *t*-test.

**Figure 6:**
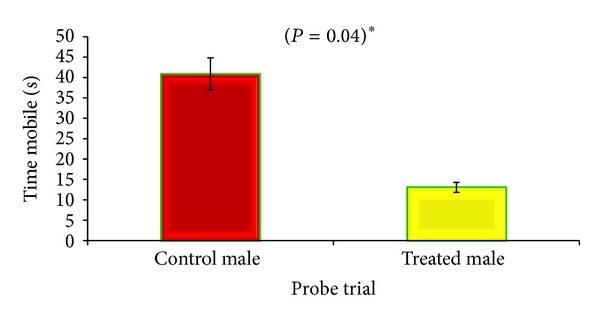
Comparison of time mobile between GABA_B_ antagonist and saline treated male albino mouse during probe trial of Morris Water Maze test. *P* value indicates the result of 2-sample *t*-test.

During probe trial, CGP 35348 treated male mice performed significantly better. They took shorter latency (*P* = 0.04) and remained less mobile (*P* = 0.04) than saline treated male as they found the hidden platform earlier than control animals (Figures 5 and 6), while none of the parameters studied in female mice reached statistical significance (data not shown here).

**Figure 7:**
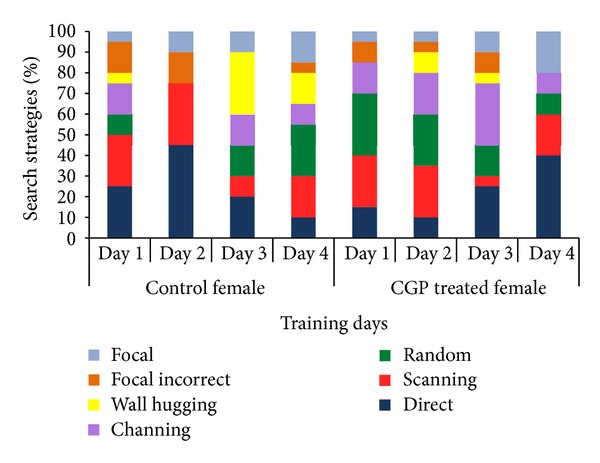
Swimming strategies of control and CGP 35348 treated female albino mouse during acquisition phase of Morris Water Maze test.

**Figure 8:**
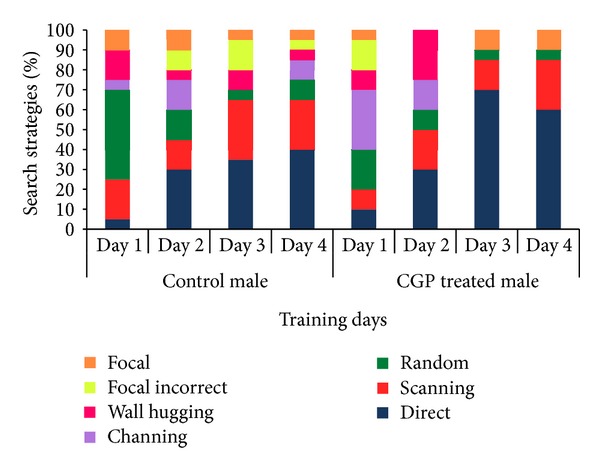
Swimming strategies of control and CGP 35348 treated male albino mouse during acquisition phase of Morris Water Maze test.

Results of swimming strategies during training days of MWM indicated that both CGP 35348 treated female and male albino mice had demonstrated improved memory formation as the direct and focal approach to the hidden platform increased, while random swimming and wall hugging decreased our the training period. Effect was more pronounced in treated male as the chaining and wall hugging strategies eliminated as the training proceeded indicating improved memory formation complementing the results of probe trial (Figures 7 and 8).

## 4. Discussion

In the central nervous system, GABA_B_ receptor regulates cyclic AMP (cAMP) levels through adenylyl cyclase activity. Activation of the cAMP regulatory pathway is vital for long-term memory formation across a variety of species [18, 19]. Recent studies indicated that metabotropic GABA_B_ receptors in the hippocampus are directly coupled to CREB-2 transcription factors which appear to serve as memory suppressors [20–22]. Removal of the repressive action of CREB-2 is thought to be mediated by protein kinase A (PKA, an intracellular second-messenger involved in long-term memory and short-term memory formation) and another protein, MAPK (catalytic subunit of PKA called mitogen-activated protein kinase) [10]. Present study was designed to suppress the GABA_B_ receptor mediated activity by using CGP 35348 (GABA_B_ receptor antagonist) and to observe its potential effect on the learning, memory formation, and neuromuscular coordination in albino mouse.

The rota rod test is widely used to determine the motor coordination in rodents [23]. It provides diverse measureable, continuous variables (time length) used for statistical purposes to appraise the effects of different conditions, procedures, and drug's effects [24]. Our results indicated no significant effect GABA_B_ receptor antagonist (CGP35348) in both male and female mice when compared with untreated controls indicating that it has no influence on neuro-muscular coordination. This observation is in agreement with [14] which has reported that GABA_B_ receptor antagonist CGP 35348 is unable to evoke measurable effects on motor performance during the rota rod experiments.

While analyzing the exploratory behavior in mice through open field test, various parameters in the open field test were considered. Our results indicated gender specific effect of CGP 35348 injections as treated females had demonstrated poor exploratory behavior as compared to saline injected females for several parameters (time mobile (*P* = 0.04), time immobile (*P* = 0.04), rotations (*P* = 0.04), and anticlockwise rotations (*P* = 0.038)) (Table 1), while there was no effect of CGP 35348 supplementation in male albino mice (data not shown here). These results are in agreement with the findings of [25, 26] which had reported that GABA_B_ receptor antagonist had very low or no effect on the fundamental mouse behaviors.

In order to test hippocampal-dependent learning, including acquisition of spatial memory and learning for albino mouse, the Morris water maze (MWM) was applied. The CGP 35348 treatment led to the improvement of learning during the acquisition phase and significantly improved memory formation during probe trail in male albino mouse as they found the platform earlier and had shorter latency as compared to the saline treated male mice (Figures 5 and 6). These results are in agreement with those of [9] who had reported that GABA_B_ receptor antagonist CGP 35348 is capable of improving learning and memory in test of cognitive functions and formation of LTP in mice.

Various doses of CGP 35348 were applied [27] by pressure ejection to one of two recording sites in area CA1 of hippocampal slices in order to observe its effect on long term potentiation (LTP) and reported that memory was enhanced at intermediate dosages but not at very low and high concentrations. Similar observations were reported in rats as they had observed that very low and high doses did not help in memory retention [27, 28]. The dose applied in present study (1 mg/mL of solvent/Kg body weight) is probably too low as only selective parameters of various studies neurological tests were affected by the application of CGP 35348.

## 5. Conclusion

We concluded that CGP 35348 has a potential to improve the various aspects of behavior in a gender specific manner in albino mice. It has improved the memory formation of male mice during retention phase of MWM, but has negatively affected the exploratory behavior of female albino mice, while the rota rod test remained unaffected in both genders. Repetition of these tests following the application of higher doses of CGP 35348 would reveal more interesting results.

## Figures and Tables

**Table 1:** Comparison of the studied open field parameters between saline and CGP 35348 treated female albino mice. *P* values indicate the results of two-sample *t*-test.

Parameters	Control female (*N* = 5)	CGP 35348 treated female (*N* = 5)	*P* value
Distance (m)	21.4 ± 5.3	11.9 ± 8.2	0.072
Mean speed (m/sec)	0.04 ± 0.01	0.02 ± 0.01	0.071
Time mobile (sec)	492 ± 26.2	296.8 ± 148.4	0.044*
Time immobile (sec)	108 ± 26.2	303.3 ± 148.4	0.044*
Mobile episodes	29.4 ± 8.0	29.6 ± 14.1	0.979
Immobile episodes	29.4 ± 7.8	29.2 ± 14.3	0.979
Max speed (m/sec)	0.3 ± 0.2	0.2 ± 0.1	0.158
Rotations	25.6 ± 6.8	13.6 ± 8.3	0.041*
Clockwise rotations	9.6 ± 6.9	6.4 ± 3.4	0.397
Anticlockwise rotation	16.0 ± 1.1	7.2 ± 5.3	0.038*

*P* > 0.05 = nonsignificant; *P* ≤ 0.05 = least significant*.
